# Physicochemical Characterization and Properties of Cassava Starch: A Review

**DOI:** 10.3390/polym17121663

**Published:** 2025-06-15

**Authors:** Andrés Felipe Chamorro, Manuel Palencia, Tulio Armando Lerma

**Affiliations:** 1 Research Group of Electrochemistry and Environment (GIEMA), Faculty of Basic Sciences, Universidad Santiago de Cali, Cali 760035, Colombia; 2 Research Group in Science with Technological Applications (GICAT), Department of Chemistry, Faculty of Natural and Exact Science, Universidad del Valle, Cali 760032, Colombia; 3 Mindtech Research Group (Mindtech-RG), Mindtech SAS, Cali 760026, Colombia; t.lerma@mindtech.com.co

**Keywords:** cassava starch, composition, physical and chemical properties, characterization methods

## Abstract

Cassava (*Manihot esculenta Crantz*) is a tuber and one of the most important sources of commercial production in the world, with high consumed food produced in the tropics contributing to high quantity of calories to the diet. The principal component of the cassava root is starch, a polysaccharide composed of amylose and amylopectin, where the physicochemical property of the biopolymer is altered in different cooking processes. This review summarizes and provides information about cassava starch structure, morphology, and physicochemical properties, such as gelatinization, pasting, and retrogradation of cassava starch, as well as the use of methods of characterization to follow and analyze the physical and chemical changes of cassava starch.

## 1. Introduction

Cassava (*Manihot esculenta*) is a tropical crop that serves as a staple food for over 800 million people. It is cultivated across a wide range of soils in tropical and subtropical regions [1,2]. In 2020, global cassava root production reached approximately 271.6 million tons, with Sub-Saharan Africa, Southeast Asia, and Latin America being the primary producers, contributing 166.0, 51.1, and 40.5 million tons, respectively [3]. This tuber is valued by various industries, including for pharmaceuticals, textiles, cosmetics, petroleum, biodegradable products, animal feed, and alcohol production [4]. The nutritional composition of cassava depends on several factors, such as soil composition, geographical location, and the age and part of the plant analyzed [5]. Cassava can grow in a variety of soils; however, light sandy loams, loamy sands, and soils with a loose structure promote better root development. Nevertheless, cassava does not thrive in alkaline soils (pH > 8) [6].

**Figure 1:**
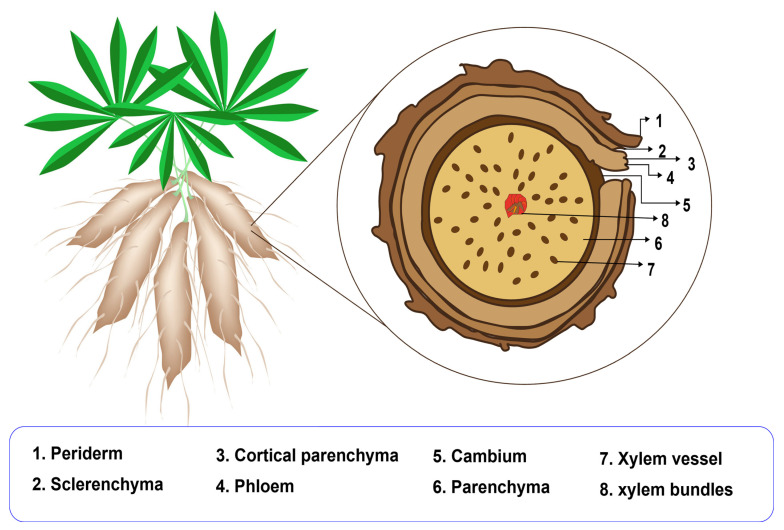
Cassava plant and cross-section of cassava roots. Adapted from [6].

Cassava plants reach maturity between 8 and 18 months and can grow up to 5 m in height [6]. Depending on the plant’s age and growing conditions, cassava roots can exhibit various shapes, including conical, conical–cylindrical, cylindrical, and fusiform, with diameters ranging from 3 to 15 cm. Carbohydrates are the main component of both cassava leaves and roots, with starch being predominant, accounting for approximately 73.7% to 84.9% of the dry weight. Small quantities of sucrose, glucose, fructose, and maltose are also present [5]. In addition, cassava is rich in calcium, potassium, magnesium, and vitamins A and C [7]. The peel of the cassava root is about 1 ± 4 mm thick and represents 10 to 13% of the total dry matter of the root [8]. It consists of two main layers: (i) the outer layer (periderm) and (ii) the inner layer (cortex), which is further divided into sclerenchyma, cortical parenchyma, and phloem tissue (Figure 1).

The main chemical components of cassava peel are carbohydrates such as cellulose, holocellulose, hemicellulose, and lignin [8]. The central region of the cassava root is protected by the cambium tissue, and within it are the parenchyma tissue and the xylem vessels. The parenchyma tissue stores high concentrations of starch, which is present in the form of granules embedded in a matrix of cassava pulp along with other constituents, such as proteins, fats, and soluble carbohydrates. Therefore, achieving efficient separation of starch granules from the cassava pulp is a key objective. To extract starch from cassava pulp, a milling machine is typically used to shred the pulp and to release the starch granules by breaking the phloem and xylem cells [9]. However, starch exists in both free and bound forms within the fibrous matrix. The separation of bound starch is more challenging, requiring mechanical and centrifugal forces. Even so, the process is often incomplete, and approximately 10% of the bound starch remains unrecovered from the cassava pulp [10]. The quality of the extracted cassava starch is commonly assessed through proximate analysis, including starch, fiber, fat, ash, and protein content. However, understanding other physicochemical properties, such as molecular structure, granular morphology, thermal behavior, and starch modifications, is crucial for evaluating and improving starch performance. Therefore, this review summarizes the physicochemical characteristics of cassava starch, with a focus on gelatinization, pasting, and retrogradation behaviors, as well as the characterization methods employed.

## 2. Cassava Starch Structure

**Figure 2:**
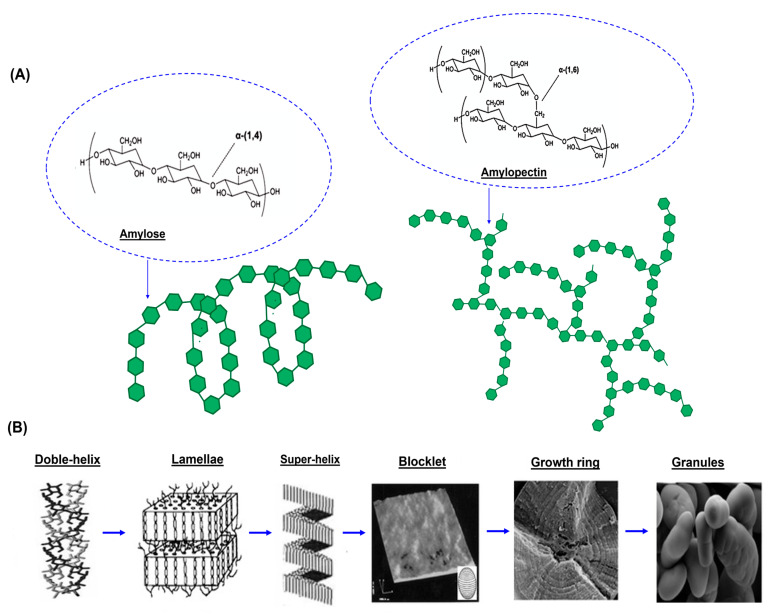
(**A**) Structure of amylose and amylopectin. (**B**) Structural organization of amylose and amylopectin in cassava starch. Adapted from [18].

Starch (C_6_H_10_O_5_)_n_ is one of the most abundant polysaccharides in nature, alongside cellulose and chitin. It is a biodegradable, biocompatible, and non-toxic biopolymer found in high concentrations in tubers, vegetables, and cereals. Starch is a copolymer composed of amylose (AM) and amylopectin (AP) (Figure 2A). Typically, starch consists of approximately 20–30% AM and 70–80% AP [11]. This polymer is biosynthesized within plant cells and stored in tubers and endosperms [12]. It aggregates into starch granules of varying sizes (2–150 µm), with their size, distribution, and shape depending on the botanical source [13]. Starch is generally insoluble in water, alcohol, and ether [6]. However, the granular surface is hydrophilic due to the presence of hydroxyl (-OH) groups, which enable strong intermolecular hydrogen bonding with solvents [14]. Structurally, AM is a long, unbranched, linear polysaccharide composed of glucose units linked by α-1,4-glycosidic bonds, with a molecular weight ranging from 10^5^ to 10^6^ g/mol [15]. It forms a three-dimensional helical structure, packed in parallel to form either A- or B-type allomorphs [16,17].

AM crystalline A has a monoclinic unit cell with dimensions a = 2.124 nm, b = 1.172 nm, c = 1.069 nm, and γ = 123.5°, consisting of 12 glucose residues and a few water molecules. By contrast, AM crystalline B exhibits a hexagonal unit cell with dimensions a = b = 1.85 nm, and contains approximately 30 to 40 water molecules per unit cell. AP is a branched macromolecular polymer composed primarily of α-(1→4)-D-glucopyranose units, with α-(1→6) branch linkages occurring approximately every 20 glucose units [19]. It has a degree of polymerization on the order of 10^4^ to 10^5^ glucose units per molecular chain [20], and branch chain dimensions in the range of 0.3 to 0.5 nm [21]. The double helix cluster model of AM is illustrated in Figure 2B. Crystalline regions are formed by the ordered packing of branched chains, while the (1→6) branch points reside in the amorphous regions [17]. AM and AP organize into alternating crystalline and amorphous lamellae with a periodicity of 9 nm, forming superhelical structures [18]. Together, they give rise to complex morphologies, such as blocklets and semicrystalline or amorphous growth rings (Figure 2B). Blocklets range in size from 20 to 500 nm; smaller blocklets contribute to amorphous growth rings, while larger blocklets form semicrystalline growth rings.

The primary use of cassava starch is as a food product due to its high carbohydrate content, making it a vital source of dietary energy for many people around the world. However, the quality of cassava starch products depends on their chemical composition. The contents of AM, protein, lipids, and fiber are the main chemical parameters that define the quality of this tuber. Table 1 presents the wide variation in the chemical composition of cassava starch. The AM content typically ranges from 13% to 29.5%, significantly influencing the functional properties of starch in food products [22]. The ranges for protein, lipid, and fiber content are approximately 0.10–4.85%, 0.08–1.59%, and 0.04–3.14%, respectively. The fiber report refers to crude fiber, which consists of organic substances that do not dissolve after hydrolysis in acidic and alkaline media. The diverse industrial applications of cassava starch depend heavily on its physicochemical properties. The following sections describe and summarize the methods and techniques used to characterize cassava starch, offering insights and recommendations for future studies involving this polysaccharide and its derivatives.

## 3. Morphology and Characterization of Physical Cassava Starch Changes

### 3.1. Cassava Starch Granule Morphology

The shape and size of starch granules are among the most important characteristics in determining starch functionality. Understanding the variation in the granule size is valuable for assessing both food quality and processing performance. According to Table 2, the most commonly reported shape is truncated; however, cassava starch samples typically contain a mixture of shapes, including oval, round, spherical, irregular, and polygonal forms [36]. The size of cassava starch granules generally ranges from 5 to 40 µm, as reported for samples obtained from crops cultivated in Thailand, Brazil, and Nigeria. The shape and size distribution of cassava starch granules are typically determined using scanning electron microscopy (SEM) and/or light microscopy. Both techniques are effective for evaluating size distribution, although SEM offers the additional advantage of analyzing granule surface morphology and porosity [37]. To obtain a more detailed surface analysis, atomic force microscopy (AFM) has been used to examine the granule surface, allowing for the visualization of blocks, depressions, and protrusions (Figure 2B).

Morphological discrepancies between different microscopy techniques, such as AFM and SEM, are commonly attributed to the inherent structural heterogeneity of cassava starch granules. Sizes reported in the literature range from 1 μm to over 100 μm. The size and size distribution depend on several factors, including botanical origin, the AM and AP content, and chemical composition, among others. These factors affect surface properties, such as pore formation, surface roughness, swelling, and overall physicochemical behavior [44,45,46]. Thus, the information obtained from SEM and AFM analyses can be correlated with the functional performance of starch in specific industrial applications. For example, in the baking industry, larger granules are preferred, as smaller granules with their higher surface area-to-volume ratio exhibit greater affinity for water, proteins, and lipids in the dough, leading to reduced dough stability and lower loaf volume [47]. In another case, for the production of biodegradable films, smaller starch granules promote higher tensile strength and reduced thickness, indicating improved film quality [48]. These examples demonstrate that SEM and AFM size data are highly valuable for the design of starch-based products; however, their relevance depends on the specific application.

On the other hand, the granule size depends on several factors, including crop age and environmental conditions. Moorthy and Ramanujam investigated the relationship between granule size and crop age using six cassava varieties, finding that the granule size increased up to the sixth month, and then remained nearly constant [44]. Other studies on three cassava varieties (CMR38-125-77, Kasetsart 50, and Rayong 11) reported that the granule size distribution continued to increase until 12 months after planting [45]. Weather conditions, especially soil temperature, also affect granule size distribution. For instance, cassava roots harvested in November (the rainy season in Colombia) showed a narrower size distribution than those harvested in January, March, or August for four cultivars (HMC-1, CM489-1, CM681-2, and CM1559-5) [27]. Excess rainfall increases water stress, which in turn reduces the starch granule size [38].

### 3.2. Molecular Mass of Cassava Starch

Starch molecular weight (M_w_) or molar mass is a key physical property in polymers, as it helps establish relationships with the physicochemical and biological properties of starch. This understanding is crucial for optimizing starch applications in various industrial sectors, particularly the food industry. The AM, AP, and M_w_ of starch can be influenced by cultivar, soil composition, and post-harvest storage conditions. For other starch sources, such as sweet potato, potato, waxy corn, and yam, it has been demonstrated that these parameters vary with the growth period [49]. Generally, for many starches, an increase in growth time leads to a decrease in the AM content, which induces changes in the granule size distribution. However, cassava starch exhibits different behavior. For example, Tan et al. (2017) evaluated the AM content and the M_w_ of cassava genotype South China 5 (SC5) harvested at 7, 8, 9, 10, and 11 months [50]. They found that the AM content decreased from 22.16 ± 0.05 % at 7 months to 20.93 ± 0.07% at 8 months, then increased to 22.61 ± 0.02% by 11 months [50]. This pattern contrasts with the findings of Sriroth et al. (1999), who reported a continuous decrease in the AM content with increasing harvest time [24]. These differences suggest that the effect of harvest time on the AM content is species or genotype dependent. Tan et al. (2017) also observed that the starch M_w_ increased with harvest time, from 0.628 × 10^7^ g/mol at 7 months to a peak of 2.640 × 10^7^ g/mol at 9 months, then decreased to 1.365 × 10^7^ g/mol by 11 months [50]. Interestingly, the highest AP content was reported at 9 months, indicating this time point as a key inflection in starch development. From 7 to 9 months, the plant undergoes developmental changes that result in a higher M_w_, suggesting that cassava starch functional properties can be modulated through control of growth duration. However, this effect is strongly dependent on plant genotype. In addition, seasonal variation also affects cassava starch properties, particularly the AM content. Teerawanichpan et al. (2008) evaluated the AM content of cassava starch from R1 and KU50 varieties grown in the wet and dry seasons [42]. For the R1 variety, the AM content gradually increased over time during the wet season, while remaining relatively constant during the dry season. By contrast, the KU50 variety showed almost constant AM levels in both seasons [42]. These results support the findings of Tan et al. (2017), indicating that the AM content in cassava starch are genotype-dependent rather than universally governed by environmental conditions [50]. Although numerous studies have evaluated the effects of environmental and soil conditions on cassava growth [24,51,52,53], and on cassava starch variability during post-harvest handling and storage [49], there is a notable gap in the literature regarding the correlation between these factors and the starch M_w_. This represents an important area for future research.

Several analytical techniques have been employed to determine the M_w_, the apparent radius of gyration (R_g_), and the apparent hydrodynamic radius (R_H_) of cassava starch. These include size-exclusion chromatography (SEC) [32], gel permeation chromatography (GPC) [54], and high-performance size exclusion chromatography (HPSEC) combined with multi-angle laser light scattering (MALLS) and differential refractive index (DRI) detection [55], and asymmetrical flow field-flow fractionation (A4F) coupled with MALLS and RI detectors [56]. Table 3 presents the dissolution methods and the corresponding values of M_w_, R_g_, and R_H_ for cassava starch reported in the literature.

All the parameters showed a wide range, demonstrating significant variability among the samples. Typically, determining the M_w_ of starch involves three main steps, including (1) complete solubilization of starch in a suitable solvent, (2) measurement, and (3) data analysis. However, solubilizing cassava starch in aqueous solutions can be challenging, as it often requires high temperatures that may cause physical alterations, such as degradation, depolymerization, and oxidation, thereby modifying its M_w_ [57]. These issues are particularly problematic in chromatographic processes, where shear forces can further degrade the starch [58]. To overcome solubility issues, water is commonly replaced with solvents such as dimethyl sulfoxide (DMSO), DMSO–lithium bromide (LiBr), or aqueous DMSO solutions [59,60]. SEC enables separation based on hydrodynamic volume rather than the M_w_. This separation principle is independent of polymer type and allows for the M_w_ estimation using standard calibrations. During analysis, the sample is injected into a column and carried by the solvent through a porous matrix. Separation occurs according to the polymer’s size relative to the pore size of the column [60].

**Table 3:** Reported average molecular weight (M_w_), apparent radius of gyration (R_g_), and apparent hydrodynamic radius (R_H_) values for cassava starch, amylopectin, and amylose from the literature.

Dissolution Process	Method	M_w_ (g mol^−1^)	R_g_ (nm)	R_H_ (nm)	Ref.
90% DMSO/H_2_O heated in boiling water-bath for 1 h with intermittent vortex mixing.	SEC/MALLS	4.76 × 10^7^ (AP) 2.52 × 10^6^ (AM)	118.6 ± 0.41	--	[32]
DMSO/LiBr heated, degassed with ultrasound in a boiling water bath for 1 h with continuous shaking.	GPC/MALLS	1–3 × 10^7^	95–135	--	[54]
95% DMSO/H_2_O and solubilised in water by microwave heating under pressure.	HPSEC/MALLS/DRI	1.30 × 10^8^	186 ± 2		[55]
DMSO/water (95/5) mixture and then solubilized in water by microwave heating under pressure.	A4F/MALLS	2.71–5.51 × 10^8^ (AP)	229–286	-	[56]
DMSO /LiBr heated at 95 °C in boiling water-bath while stirring for 15 min, and 18 h at room temperature while continuously stirring.	SEC/MALLS	5.7 × 10^7^	--	283–138	[59]
90% DMSO and heated in a boiling water bath for 1 h with constant stirring.	HPSEC/MALLS/DRI	2.0 × 10^8^	171.1 ± 18.5	-	[61]
Starch samples were heated in a boiling hot water bath for 20 min with continuous stirring.	HPSEC/MALLS/R	2.72 × 10^8^	171.2 ± 3.67	-	[62]
Water by microwave heating under pressure.	HPSEC/MALLS/VS/DRI	4.10 × 10^7^–2.17 × 10^7^	130–132	89–97	[63]
Sample solved in DMSO with concentration between 1–3 g mL^−1^.	SLS	1.0 × 10^8^	-	-	[64]
Starches (25 mg) were solubilized in 1 M KOH (0.5 mL) for 3 days at 4 °C under gentle magnetic stirring, and 4.5 mL pure water and 0.1 M HCl (5 mL).	A4F/MALLS and HPSEC/MALLS	4.08 × 10^8^–5.20 × 10^8^	277–285	-	[65]

Despite the widespread use of SEC for polymer analysis, determining the M_w_ of starch remains difficult. The AP component is particularly susceptible to shear scission, making it nearly impossible to obtain reliable size distributions for this fraction using conventional SEC methods [58]. In addition, there are few available calibration standards with molar masses in the same range as those of starches, which makes accurate measurement challenging. Nevertheless, SEC has been used to determine the M_w_ distribution of both fully branched and debranched cassava starch, revealing molecular size distributions ranging from 1 to 200 nm. Two distinct molecular populations were identified, corresponding to hydrodynamic radii of approximately ~20 nm (AM) and ~90 nm (AP) [66]. Gel permeation chromatography (GPC) coupled with MALLS has also been employed to evaluate the effect of growth time on the M_w_ and R_g_ of cassava starch. Both parameters showed a significant increase, with the M_w_ rising from 1.8 × 10^7^ to 3.3 × 10^7^ g·mol^−1^, and the R_g_ from 95 to 135 nm, as the crop’s growth period extended from 7 to 10 months [54].

The limitations observed with SEC are addressed by using HPSEC, which integrates high-performance liquid chromatography (HPLC) into the SEC system [67]. HPSEC coupled with MALLS and a DRI detector has been widely employed to determine the M_w_, size, and the structure of AP and starch from various sources, such as rice [68], corn [69], and potato [70]. One major advantage of MALLS is that it does not require calibration with the M_w_ standards to determine column elution volumes, unlike DRI detection [60]. Yokoyama et al. (1998) utilized HPSEC/MALLS to determine the M_w_ of cassava starch dissolved in DMSO with 50 mM LiBr [59]. The average M_w_ was calculated using three extrapolation methods, including Berry, Zimm, and Debye, yielding values of 56.3, 82.9, and 48.4 × 10^6^ g·mol^−1^, respectively. Moreover, measurements using both MALLS and DRI detectors have been reported. The MALLS profile typically displays a single peak corresponding to the AP fraction, while DRI detection reveals two peaks corresponding to the AP and AM fractions, with elution volumes of 5.45 mL and 6.14 mL, respectively. Using this approach, cassava starch was found to have a M_w_ of 1.30 × 10^8^ g·mol^−1^ and an R_H_ of 186 ± 2 nm [55]. Additionally, the AP molar mass has been used to differentiate between three Ugandan cassava varieties (Bamunanika, NASE 10, and TME14), with M_w_ values of 2.56, 2.42, and 2.74 × 10^8^ g·mol^−1^, respectively, and R_g_ between 170 and 176 nm. The polydispersity index (PDI) ranged from 1.10 to 1.18 [62].

**Figure 3:**
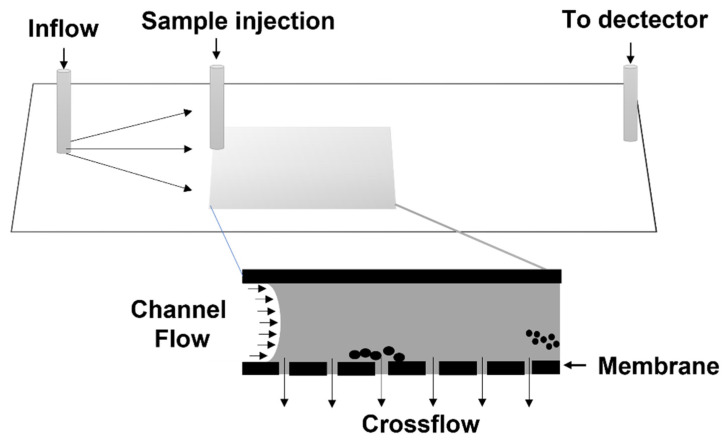
Principle of macromolecule separation using asymmetrical flow field-flow fractionation (AF4).

Another technique used to determine the M_w_ of cassava starch is AF4 coupled with MALLS and RI detectors (AF4/MALLS/RI). This technique enables the characterization of solutes with the M_w_ ranging from ~10^3^ to ~10^9^ g·mol^−1^ [60,71]. AF4 separates polymers based on differences in their diffusion coefficients and retention times within a laminar flow field (Figure 3). A perpendicular crossflow is applied to the channel flow, which forces solutes to different equilibrium positions. Separation occurs due to differences in the translational diffusion coefficients (D_t_), allowing for the calculation of the R_H_ under the assumption of spherical geometry, using appropriate mathematical models. The separated fractions can be analyzed by MALLS at multiple scattering angles. Using Berry, Zimm, or Debye extrapolation methods, the M_w_ of the polymers can be accurately calculated [72]. Rolland-Sabaté et al. (2013) applied AF4 coupled with MALLS to determine the M_w_ and size distribution of AP in cassava starches with low AM content obtained through transgenesis [57]. The reported M_w_ values ranged from 178 to 233 × 10^6^ g·mol^−1^, with the corresponding R_H_ values between 203 and 277 nm. These M_w_ values were significantly higher than those reported for waxy maize and waxy potato AP, which were 40.8 × 10^6^ and 52.0 × 10^6^ g·mol^−1^, respectively [65]. AF4/MALLS/RI also enables the determination of the R_g_ of cassava starch components. Juna and Huber (2012) used this method to separate the starch into AM-type (Fraction A) and AP-type (Fraction B) components by using 1 M KSCN and high-pressure microwave-assisted extraction [72]. The average M_w_ for cassava starch and the AM and AP fractions were found to be 59 × 10^6^, 21 × 10^6^, and 19 × 10^6^ g·mol^−1^, respectively, with the corresponding average R_g_ values of 165, 73, and 87 nm, respectively.

### 3.3. Starch Gelatinization

**Figure 4:**
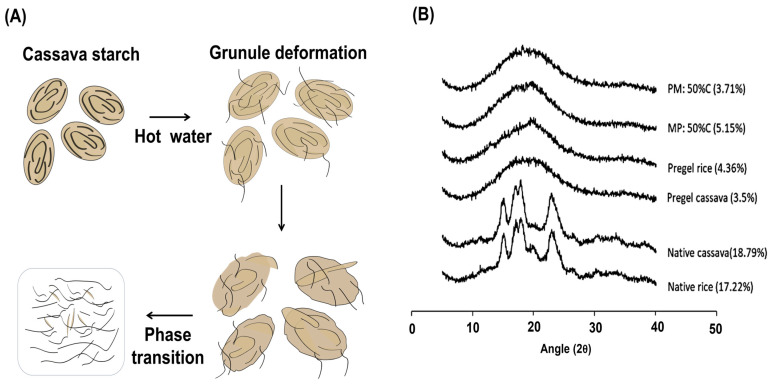
(**A**) Mechanism of starch gelatinization. (**B**) X-ray diffractograms of native cassava and rice starches, pregelatinized cassava and rice starches, and 50% pregelatinized starch blends. Adapted from [86]. MP and PM represent starch blends composed of cassava starch (50% *w*/*w*) and rice starch (50% *w*/*w*), prepared before (MP) and after (PM) pregelatinization, respectively. The percentages shown in the legend for all samples correspond to their degrees of crystallinity.

The gelatinization process of starch occurs when starch granules are heated in the presence of water at temperatures above approximately 80 °C, at which point cassava starch granules swell and progressively lose their birefringence. Starch gelatinization is influenced by several factors, including its physical and chemical characteristics, starch-to-water ratio, pH of the solution, and the presence of swelling-promoting salts, fats, and fiber [73,74]. This process occurs in three main stages as follows:

(i) Initial hydration: Water is reversibly adsorbed on the surface of the granules, causing them to swell.
(ii) Granule expansion and chain release: As more water is absorbed, the granules significantly increase in volume, which leads to a rapid rise in system viscosity and loss of birefringence. Simultaneously, the AM and AP chains are released and form a viscous solution through hydrogen bonding with water [73]. Gelatinization is thus a physical process that begins in the amorphous regions of the granule, where intermolecular forces are relatively weak [75]. This process results in the irreversible loss of molecular order, representing an order–disorder phase transition. From a thermodynamic perspective, it corresponds to an increase in the configurational entropy of the polymer chains (Figure 4A) [73].
(iii) Granule disintegration: As the system surpasses the critical temperature, the granules lose their structural integrity, becoming formless sacs that disintegrate and disperse in hot water [76].

Each granule gelatinizes at a specific melting temperature, so the gelatinization of a starch population occurs over a temperature range [74]. According to small-angle light scattering measurements, Marchant and Blanshard (1978) demonstrated that the loss of birefringence is associated with polymer solvation and a structural transition from a helical to a random coil conformation [77]. After reaching the critical temperature, and upon cooling, the polymer chains undergo molecular reassociation, leading to the formation of new hydrogen bonds and intermolecular interactions [78]. On the other hand, the gelatinization process is influenced by the heating rate. Generally, gelatinization transition temperatures increase as the heating rate increases. Additionally, higher heating rates tend to intensify the endothermic transitions, which are attributed to the presence of various crystallite structures. At elevated heating rates, a temperature gradient is established within the sample, leading to a broadening of the endothermic peak [79]. This temperature gradient affects the interaction between water and starch polymer chains through hydrogen bonding. As a result, the disordering of the crystalline regions within the granules and the leaching of soluble polymer chains are impacted by the rate of heating.

Several methods and techniques have been used to determine the critical temperature and monitor the starch gelatinization process, including X-ray diffraction (XRD), differential scanning calorimetry (DSC), loss of turbidity, and enzyme-catalyzed hydrolysis rate. In the case of cassava starch, gelatinization can be followed using X-ray diffraction patterns before and after the thermal process. There are three main crystalline forms of native starch, identified by their characteristic XRD patterns. The A-type crystalline structure exhibits characteristic XRD peaks at 2θ values of 15°, 17°, 18°, and 23°, corresponding to left-handed, parallel-stranded double helices packed in a monoclinic unit cell. The B-type structure, commonly found in potato starch, shows peaks at 5.6°, 15°, 17°, 22°, and 24°, and consists of six left-handed, parallel-stranded double helices arranged in a loosely packed hexagonal unit cell [80]. The third type, C-type, presents peaks around 5.6°, 15°, and 23°, and is considered a hybrid structure composed of B-type crystalline regions surrounded by A-type crystallites. While the A-type crystalline structure is most commonly reported for cassava starch [81,82], both C-type [83] and B-type [84] have also been observed. The degree of crystallinity significantly affects the gelatinization temperature. Higher crystallinity is associated with elevated gelatinization temperatures, due to stronger interactions between the crystalline and amorphous regions of the starch granules [85]. Additionally, greater crystallinity enhances the mechanical activation of starch, which refers to structural modification induced by mechanical forces, a process widely applied in the production of starch-based materials [83].

In some studies, this transformation is referred to as “melting” to describe the disruption of crystalline regions [86]. Before gelatinization, X-ray patterns show distinct crystalline reflections associated with crystal planes. After the so-called “melting,” these reflections disappear, indicating a transition from a crystalline to an amorphous state [87]. This transition is evident in the XRD patterns of cassava and rice starches in their native, partially gelatinized, and fully gelatinized forms (Figure 4B). Both starches initially show an A-type crystalline pattern. After gelatinization, the pregelatinized samples display XRD patterns without sharp diffraction peaks, clearly differentiating them from native starches. This indicates that the semi-crystalline structure has been disrupted and converted into an amorphous phase [86,88].

DSC has been widely used to determine the heat capacity change associated with water–starch interactions and the phase transitions of the system induced by temperature, reflecting the energy absorbed during the transition [89]. This technique enables (i) the study of gelatinization at various starch–water ratios, (ii) the determination of gelatinization temperatures above 100 °C, and (iii) the calculation of the enthalpy change (ΔH) of the transition process [90,91]. When starch is heated in the presence of water to its glass transition temperature (T_g_), a phase transition begins. However, the energy absorption peak associated with T_g_ is usually not evident in DSC thermograms. This is because the melting of the crystalline region, which may comprise up to 30% of the total starch granule follows T_g_ very closely. Consequently, the endothermic peak corresponding to melting (T_m_) has a much larger enthalpy than that of the glass transition [73]. This transition behavior is influenced by intermolecular interactions within the cassava starch chains. For instance, Beninca et al. (2013) reported DSC thermograms for both native cassava starch and starch treated with different concentrations of NaClO, an oxidizing agent [92]. In these treated samples, the hydroxyl groups of the starch were partially converted to carboxyl groups, resulting in new molecular interactions. The study found that the gelatinization temperature increased with increasing NaClO concentration, due to the formation of carboxyl and carbonyl groups, which strengthen molecular interactions and modify the physicochemical properties of cassava starch.

Table 4 presents the gelatinization temperatures of various starches obtained from different cassava crops. The temperature range varies from 69.15 to 85.1 °C, and the gelatinization enthalpy ranges from 4.8 to 17.4 J g^−1^ for these samples. This wide variation suggests that cassava starch gelatinization is influenced by several factors, including environmental conditions, agronomic practices, and processing parameters. For instance, the effect of environmental conditions on cassava starch gelatinization was investigated by Defloor et al. (1998) using the DSC analysis of four cassava genotypes harvested at 6, 9, 12, 15, and 18 months after planting [85]. The gelatinization temperatures varied between 62 and 76.1 °C, and enthalpy values ranged from 9.0 to 15.0 mJ g^−1^ (dry matter basis). The study concluded that both genetic factors and environmental conditions significantly affect starch gelatinization behavior [85]. Although there is limited information on how environmental conditions affect the gelatinization temperatures of cassava starch, studies on other starch sources, such as wheat and rice, have demonstrated that these effects are largely influenced by the crystalline structure. For example, in wheat, temperatures above 30 °C during grain maturation lead to a higher proportion of A-type starch granules in the endosperm, suggesting that heat stress impacts starch granule formation [93]. Furthermore, temperature influences the structural development of starch granules. Liu et al. (2017) found that increasing temperature affects the granule size distribution, resulting in larger diameters, volumes, and surface areas [94]. This, in turn, causes an increase in gelatinization temperature because larger granules typically exhibit a more ordered and crystalline structure, requiring more energy to disrupt the crystallites and enable hydration of the AM and AP components [94].

DSC has also been applied to determine gelatinization temperature and enthalpy after starch hydrolysis using commercial fungal enzymes, such as α-amylase and glucoamylase. Enzymatic hydrolysis is employed to produce porous starch granules with improved properties, including enhanced hydration and thermal behavior [102]. Cornejo et al. (2022) reported that, following enzymatic hydrolysis, the gelatinization temperature increased while the enthalpy of gelatinization decreased for INIAP 650 and INIAP 651 starch samples [102]. However, these results vary in the literature, reflecting the complexity of enzymatic hydrolysis, which depends on multiple factors, such as enzyme type and concentration, reaction time, and starch species. Consequently, differing trends in the physicochemical properties of starch after hydrolysis represent a gap in the literature. Some researchers attribute the increase in gelatinization temperature to the restricted swelling caused by amylose–lipid complexes, which limit granule expansion during gelatinization [103]. Others suggest that this increase arises from a reorganization within the granule structure, where the amorphous regions become glassier [104].

Among environmental factors, humidity plays a particularly critical role in the gelatinization process. Garcia et al. (1996) studied the thermal transitions of cassava starch with water contents ranging from 35 to 60% *w*/*w*, observing that, at high water content, gelatinization occurred in a single stage, while, at intermediate water content (45%), a two-stage melting process was observed [105]. This behavior indicates that starch granules respond differently to moisture levels. When high humidity is present, the gelatinization transition is more complete [105]. By contrast, limited water results in a two-stage transition: the first stage involves partial melting of the crystalline regions, and the second stage involves a higher-temperature endothermic peak, which corresponds to the melting of the remaining crystalline structures [75,106]. Thus, increasing moisture availability in starch granules facilitates the gelatinization process. These findings have potential agronomic applications. For instance, increased irrigation during crop development could reduce the degree of molecular packing within starch granules, thereby lowering the energy required for gelatinization [107].

Starch can be classified nutritionally into three categories: (i) rapidly digestible starch, (ii) slowly digestible starch, and (iii) resistant starch. Resistant starch, which refers to starch that is not absorbed in the small intestine of healthy humans [108], is generally correlated with high AM content in the granule, which varies depending on the plant species [109]. It also depends on factors such as branch chain length distribution and crystalline structure type [110]. However, there is limited information on the resistant starch in cassava and, to our knowledge, no studies have reported correlations between environmental conditions and the resistant starch content in cassava starch. Mejía-Agüero et al. (2012) analyzed 25 cassava varieties cultivated under uniform conditions, and evaluated the resistant starch in 10 randomly selected samples [111]. They found resistant starch levels ranging from 85 to 196 g/kg (dry basis). The samples exhibited a C-type crystalline pattern by XRD, which is associated with slow and incomplete digestion [111].

### 3.4. Pasting

Before the gelatinization transition is complete, continuous heating and shear conditions cause the granules to become formless sacs, eventually disintegrating and dispersing into the hot water to form a paste. This is a physical process that follows gelatinization, in which the AM and AP polymers are dispersed into the aqueous medium. The resulting system consists of a discontinuous phase made up of swollen granules (primarily composed of AP) and granule fragments, forming a viscous mass. Upon cooling, this system transforms into a viscoelastic gel [73]. This viscoelastic gel is formed through molecular interactions between water and the polymer chains. Water acts as a plasticizer within the solution, promoting greater flexibility of the starch chains and enabling the rearrangement of the polymers. These rearranged chains form new intermolecular interactions and hydrogen bonds with water molecules located between the polymer layers [90]. During this pasting phase, the starch undergoes changes that result in a decrease in viscosity and swelling capacity. These effects arise due to alterations in both the amorphous and semi-crystalline regions of the starch granules [112].

**Figure 5:**
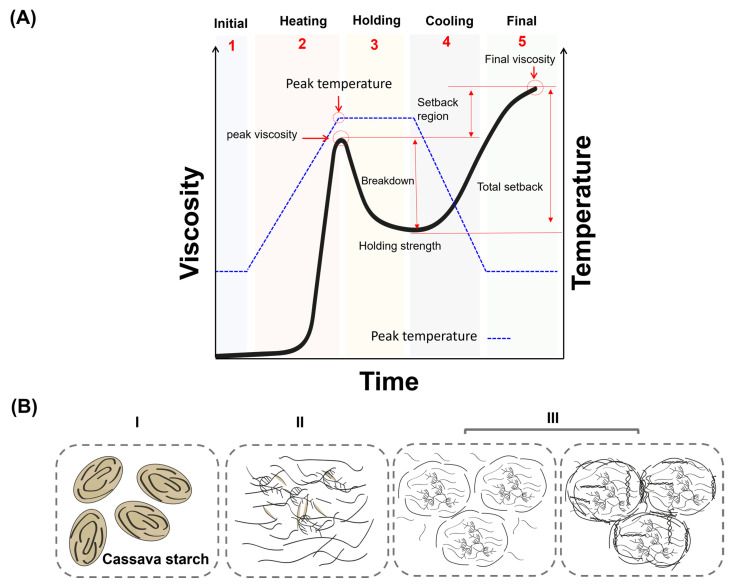
(**A**) Typical Rapid Visco Analyzer (RVA) profile of the pasting phenomenon of cassava starch. (**B**) Mechanism of starch changes in water: (**I**) Native starch granules, (**II**) Gelatinization, (**III**) Cooling, and (**III**) Retrogradation.

Recently, the pasting properties of cassava starch have been measured using the Rapid Visco Analyzer (RVA), a viscometer that allows real-time viscosity measurements while the sample is continuously stirred [113]. The RVA offers several advantages, including good reproducibility, versatility, rapid analysis, and the ability to use small sample sizes (as little as 4 g, compared to 65 g required by an amylograph) [114]. The results are reported in standard viscosity units (mPa·s). The RVA test follows a standard profile consisting of five stages (Figure 5A): (i) hydration of the sample; (ii) heating (pasting); (iii) holding at high temperature; (iv) cooling; (v) final holding at constant temperature [115]. During stage ii, it is possible to determine the pasting temperature, peak viscosity, and peak temperature of the starch. During stage v, the final viscosity is measured, corresponding to the end of the pasting cycle. This final viscosity tends to increase as the temperature decreases, due to enhanced molecular interactions among the starch polymers [115]. The pasting behavior of cassava starch in the RVA is highly dependent on operational conditions. For instance, the pasting temperature increases with a faster heating rate, and is inversely proportional to the stirring speed [116]. The viscosities of cassava starch also depend on the granule size. Hossen et al. (2011) determined the RVA parameters for cassava starch granules of four different sizes (16.6 μm, 9.7 μm, 7.1 μm, and 5.6 μm) obtained by pulverization of the same starch sample [117]. They found that both peak and final viscosities decreased as the granule size decreased, indicating that smaller particles are more resistant to swelling [117]. A similar trend was observed by Singh et al. (2010) for wheat starch, suggesting that this behavior is more influenced by the granule size than by the starch source [118]. Additionally, there is a relationship between crystallinity and the granule size, as A-type starch granules generally tend to be larger than B- and C-type granules. This difference affects gelatinization since the process is strongly influenced by the particle size. Consequently, suspensions containing A-type granules exhibit higher viscosity than those formed with other crystalline structures [118].

The pH of the aqueous medium significantly influences the rheological properties of cassava starch pasting. At pH 3 and pH 7, cassava starch exhibits different viscosity–time profiles. Under acidic conditions (pH 3), the starch undergoes partial degradation, resulting in tighter chain packing, reduced water absorption, lower swelling, and decreased viscosity. Conversely, at alkaline pH levels (above 7), the viscosity decreases progressively as the pH increases from 7 to 11 [112]. Ambient environmental conditions also play a significant role in the pasting behavior of cassava starch. Zhang et al. (2020) investigated cassava flours from seven different locations in China and reported that low ambient temperatures, particularly during the tuber maturation stage, may result in higher starch content, which contributes to greater peak viscosity and breakdown values [35]. Additionally, precipitation levels may enhance starch accumulation in cassava tubers, further contributing to higher peak viscosity in the resulting flour [35]. Although the RVA has been widely used to analyze pasting behavior, it has methodological limitations when compared to industrial processes. For example, the RVA operates under controlled temperature and standardized shear rates, conditions that often differ significantly from those in industrial settings, resulting in different pasting behaviors [119]. Additionally, the RVA profile can be influenced by other components present in the samples, such as lipids, proteins, and salts. These factors are frequently overlooked in RVA analyses, despite their impact on industrial samples [120]. Moreover, samples with varying compositions, botanical origins, and environmental factors can produce similar RVA profiles due to the complex structure of starch, which complicates data interpretation and limits the applicability of this technique [119,121]. Regarding temperature operations, the RVA instruments are limited to temperatures below 100 °C and moderate shear rates, preventing the simulation of high-temperature starch processing conditions used in industry, such as sterilization and extrusion [122]. This limitation restricts an understanding of starch behavior under these critical industrial conditions.

### 3.5. Retrogradation

Figure 5B illustrates the structural transformation of cassava starch granules during heating in the presence of water. Steps I and II represent the transition from native cassava starch granules to a gelatinized state, culminating in the formation of starch paste. Following gelatinization, step III corresponds to the retrogradation process, which occurs during cooling of the gelatinized starch–water system. During this stage, polymer chains reassociate, forming more ordered structures [18]. This reassociation promotes expulsion of the water molecules and induces partial and rapid recrystallization. Specifically, short branches of AP may form B-type polymorphic structures, while the AM chains tend to align into double-helical conformations through hydrogen bonding between glucose units [95]. As a result, several physicochemical properties of the system are altered, including viscosity, turbidity, gel-forming capacity, and crystallinity [18]. These molecular and structural transitions have been characterized using various techniques, including thermal analysis (DSC, TGA), rheological methods (e.g., RVA), XRD, and microscopy (e.g., TEM, AFM, SEM). Among these, rheological tests and XRD have proven particularly effective in investigating retrogradation, which involves recrystallization processes that modify molecular interactions and rheological properties. For instance, Rodríguez-Sandoval et al. (2008) studied cassava starch retrogradation using wide-angle XRD [123]. Native cassava starch exhibited an A-type diffraction pattern, with prominent peaks at 2θ ≈ 15.37°, 17.37°, 18.7°, and 23.37° [123]. By contrast, the retrograded starch displayed a B-type pattern, indicative of increased structural rigidity and phase separation between the polymer and the surrounding medium.

Retrogradation is a complex phenomenon influenced by multiple factors, including the starch source, the AM concentration, pH, and the cooking and cooling temperatures [123]. Among these, the AM content plays a central role, as it is widely regarded as a major driver in accelerating the retrogradation process. AM tends to rapidly form ordered matrices, which act as nucleation sites for AP, thereby promoting its slow crystallization. Indeed, AP gelation occurs at a significantly slower rate compared to AM [123]. Thus, the AM-to-AP (AM/AP) ratio in starches is a critical factor in the degradation and retrogradation processes. Starches with high AM content tend to retrograde more easily than those with lower AM levels. This behavior is related to the formation of ordered crystalline structures by AM, which can be analyzed using XRD [1]. The role of AM in facilitating retrogradation is attributed to its tendency to reassociate rapidly, contributing to the initial hardening of the gel. The AM chains form insoluble helices, which are later followed by AP recrystallization through intra- and intermolecular double helices, resulting in gel formation [124]. This effect has been widely documented in starches from rice, maize, and potato [125]. However, there is limited information in the literature specifically addressing this phenomenon in cassava starch, representing a knowledge gap that warrants further investigation.

On the other hand, water content also has a significant impact on starch retrogradation by influencing AP crystallization. Using DSC, Zeleznak and Hoseney (1986) found that the maximum enthalpy of retrogradation (~1.8 cal g^−1^) in starch gels occurs at water contents between 40 and 45% [126]. Similarly, Zhou et al. (2011) reported that the presence of 25% AM notably affects AP crystallization at higher water contents (70–80%) [127]. However, at lower water content (~60%), AP crystallization appears unaffected by the presence of AM [127].

Additives such as carbohydrates, salts, proteins, lipids, and other food-grade compounds can be used to retard starch retrogradation. The effectiveness of each additive depends on the type of polysaccharide and the source of starch. For instance, xanthan gum has been shown to enhance the retrogradation of wheat starch [128], but inhibit the retrogradation of cassava starch [129]. Several other carbohydrates, including glucose, fructose, sucrose, maltose, and carboxymethyl cellulose, have also been reported to inhibit cassava starch retrogradation. The mechanisms underlying this inhibition are primarily related to the competitive and interactive effects between the additives and starch molecules. These compounds can interact with the polymeric chains of gelatinized starch, helping to stabilize the amorphous and entangled matrix through the formation of hydrogen bonds [130]. Furthermore, carbohydrates reduce the availability of water molecules to interact with starch chains, thereby limiting the extent of retrogradation [131].

Salts, particularly sodium chloride (NaCl), are commonly incorporated into starch formulations to enhance their physical properties. In cassava starch, NaCl influences both gelatinization and retrogradation processes due to its ability to interact with the hydroxyl groups of starch chains via electrostatic interactions. Thirathumthavorn and Trisuth (2008) investigated the effect of NaCl on the retrogradation of cassava starch using DSC [132]. Their results showed that the addition of NaCl significantly decreased the retrogradation enthalpy of amylopectin-rich starch samples (*p* < 0.05). Notably, a 10% NaCl addition was more effective than 5% NaCl in inhibiting retrogradation. This effect is likely due to NaCl competing with starch molecules for water, thereby retaining water within the gel matrix and limiting the mobility and realignment of starch chains during storage. Additionally, the electrostatic interactions between the sodium (Na^+^) and chloride (Cl^−^) ions, the hydroxyl groups of the starch, and the water molecules likely prevent the formation of the ordered structures required for retrogradation.

## 4. Spectroscopic Characterization of Cassava Starch

The chemical composition and molecular behavior of cassava starch are crucial characteristics that must be determined to fully understand its physicochemical properties. This knowledge is essential for evaluating and enhancing the performance and applicability of cassava starch in both industrial and household settings. Several analytical techniques are commonly employed to characterize its functional groups, chemical structure, and chemical modifications, including Fourier-transform infrared spectroscopy (FTIR), nuclear magnetic resonance spectroscopy (NMR), and Raman spectroscopy.

### 4.1. Fourier-Transform Infrared Spectroscopy (FTIR)

**Figure 6:**
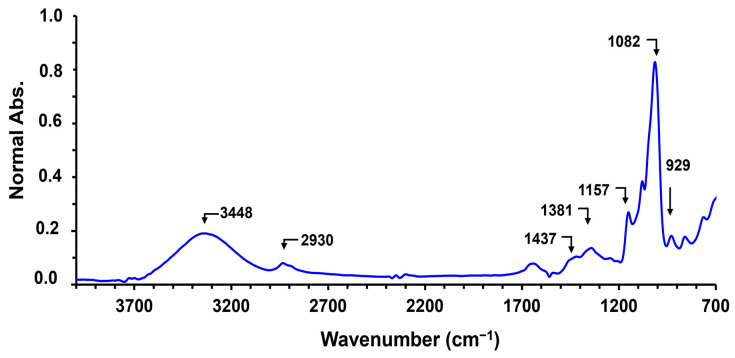
Principal bands of the ATR–FTIR spectrum of cassava starch. The cassava starch sample was obtained from a local market in Cali, Colombia. Spectrum adapted from [138].

When used in combination with attenuated total reflectance (ATR), FTIR facilitates the detection of molecular vibrations, producing characteristic infrared absorption bands associated with fundamental vibrational modes (e.g., stretching, asymmetric bending) of functional groups [133,134]. In the infrared spectrum, well-established spectral regions correspond to specific functional groups, enabling the identification of chemical functionalities present in cassava starch. These general regions include (i) the X–H stretching region (4000–2500 cm^−1^), (ii) the triple-bond region (2500–2000 cm^−1^), (iii) the double-bond region (2000–1500 cm^−1^), and (iv) the fingerprint region (1500–600 cm^−1^) [134,135]. Table 5 presents the FTIR bands reported in the literature for cassava, corn, and potato starches, while Figure 6 displays the FTIR spectrum of cassava starch. The absorption bands observed between 1700 cm^−1^ and 900 cm^−1^ are attributed to glucose units, and the monomeric building blocks of AM and AP [136,137]. When comparing the three different starches, the research did not observe significant differences in the wavenumber positions of the functional group bands. However, physical differences such as morphology and granule size were noted between the three starches. Furthermore, the infrared absorption bands observed are consistent with those reported in the other studies using FTIR to characterize starch [138,139,140,141,142].

Normally, in the FTIR spectrum of cassava starch, a band around 1640 cm^−1^ appears, attributed to the water present in the starch, which is absorbed due to its high hydrophilicity [137,143,144,145]. The FTIR results of cassava starch under thermal stress (100 °C) have demonstrated a displacement of the water signal, due to the destruction of crystalline domains and the formation of amorphous domains, affecting the environment of the water molecules, which are not completely removed by heating [137]. FTIR has also been used to characterize the chemical modifications of cassava starch, including chemical cross-linking [135,146], cationization [147], oxidation [99], acetylation [148], and hydroxypropylation [149], among other modifications. However, in many cases, spectral signal overlap makes it difficult to analyze and identify the functional groups and chemical modifications in the cassava starch structure. To address this, mathematical treatments, such as spectral deconvolution and spectral differentiation, have been applied to evaluate the overlapping signals [150]. For instance, to understand the effect of thermal stress on the starch raw material, functional-enhanced derivative spectroscopy (FEDS) was applied, demonstrating small changes in signal intensity within the wavenumber range between 3800 and 3000 cm^−1^ [137]. Data deconvolution have enabled the extraction of absorbance intensity from the glucose bands (1700 and 900 cm^−1^) to evaluate the retrogradation process [151,152]. Furthermore, deconvolution of the FTIR spectral band has been useful in visualizing the chemical cross-linking of cassava starch after treatment with plasma [153], the ozone oxidation of cassava starch in aqueous solution [154], and the esterification of cassava starch with citric acid [155], among other modifications.

On the other hand, the FTIR spectroscopy measurements have been used to determine the quantification of cassava starch, combined with chemometric tools. For example, the quantitative analysis of tapioca starch was evaluated as a method to quantify starch, protein, water, and ash using chemometric partial least squares. The method obtained a root mean square error of prediction of 0.003924% for protein, 0.3557% for water, 0.00392% for ash, and 2.3162% for starch [134], making it an alternative to replace volumetric methods [156]. Additionally, the conformational changes of the starch structure with temperature have been monitored using FTIR. Researchers have reported that the ratio of the absorbance bands between 800 and 1300 cm^−1^, corresponding to the C–O–C and C–O vibrations, can be used to follow the gelatinization and retrogradation processes of starch, as these bands are sensitive to the polymer conformation [157]. The intensity ratio of the C–O band stretching around 1082/1016 cm^−1^ is particularly sensitive to changes in starch structure [152]. Variation in absorption intensity has been associated with conformational changes: when the ratio decreases, the organization structure (gelatinization) decreases, and when the ratio increases, the organization structure (retrogradation) increases [152]. The FTIR absorbance ratio mentioned allows for an investigation of the effects on cassava starch retrogradation, such as the impact of temperature. It was found that retrogradation at −20 °C was slightly greater than at 5 °C [158]. Low molecular sugars (sucrose, glucose, and trehalose) affect the retrogradation process in starch-based foods. Analyzing the intensity ratio (1047/1022 cm^−1^) showed an increase with the addition of sugars in the order of trehalose > sucrose > glucose, suggesting that trehalose is more effective at retarding retrogradation. This behavior is attributed to the strong interaction between trehalose and starch, which stabilizes the amorphous phase and inhibits the crystallization of the starch polymer [152].

FTIR can also be used to distinguish starch from non-digestible polysaccharides, such as cellulose and inulin. Vázquez-Vuelvas et al. (2020) conducted a qualitative comparison of starch with cellulose, inulin, and agavin, identifying the region from 1200 to 900 cm^−1^, known as the polysaccharide fingerprint region, as particularly informative [159]. Although the spectra showed substantial overlap, distinct wavenumbers were observed within the fingerprint region due to differences in the stretching vibrations of C–O–H and C–O–C bonds, allowing for qualitative differentiation of these biopolymers [159]. However, when analyzing feed samples, distinguishing these polysaccharides requires statistical tools. While univariate statistical analysis has been used to differentiate between structural carbohydrates (e.g., cellulose) and nonstructural carbohydrates (e.g., starch), it is generally insufficient for precise classification. Therefore, multivariate statistical methods, such as cluster analysis (CLA) of the FTIR spectra, have been employed to improve discrimination [160]. Mid-infrared spectroscopy has also been applied to identify modified starches, using advanced data treatment techniques such as quadratic discriminant analysis, artificial neural networks, and soft independent modeling of class analogy. These approaches were successfully applied to classify 232 starch samples according to their type of modification, demonstrating the effectiveness of multivariate methods in starch analysis [161].

### 4.2. Nuclear Magnetic Resonance (NMR) Spectroscopy

NMR spectroscopy has been used for the analysis of various raw materials, including cereals, vegetables, and fruits, as sources of carbohydrates, fats, and oils [78]. NMR is a non-invasive, non-destructive technique that requires only small amounts of sample [162]. This technique involves applying a magnetic field to a sample, which induces an energy exchange between two energetic levels in resonance with the nuclear spin [163]. NMR has proven to be an important tool for characterizing cassava starch and its source (cassava). For instance, the nutrient composition of cassava varies among different cassava genotypes. Experiments using ^13^C cross-polarization/magic-angle spinning nuclear magnetic resonance (^13^C CP/MAS NMR) were conducted to investigate six cassava genotypes, revealing that each genotype exhibited distinct dynamic molecular behavior [164]. Additionally, this technique has been used to explore how harvest conditions affect the functional behavior of cassava starch. Reports using ^1^H NMR on samples subjected to thermal gelatinization and acid hydrolysis, harvested during different weather conditions (rainy or dry seasons), found that irrigation leads to a looser packing of the AM and AP chains, which promotes plasticization and gelatinization of the starch granule [107].

**Figure 7:**
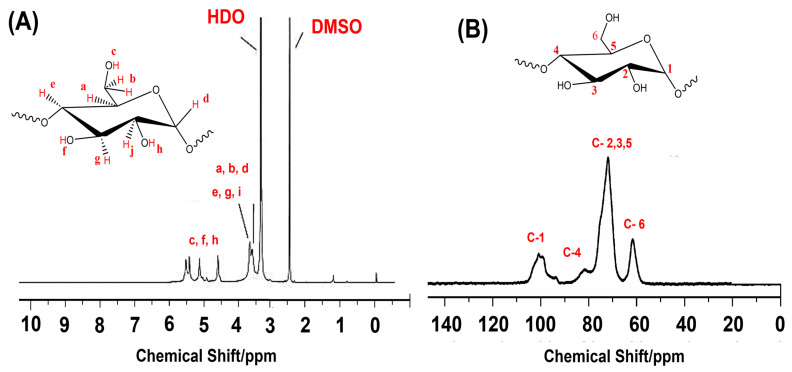
(**A**) Proton-NMR spectrum of cassava starch recorded in d6-DMSO as the solvent at 100 °C (400 MHz), adapted from [165]. (**B**) Carbon-13 CP/MAS NMR spectra of native cassava starch, adapted from [166].

For cassava starch, ^1^H NMR and ^13^C NMR have been used in both solid and liquid states to study the starch chemical composition and chemical structure (Figure 7). They have also been used to monitor structural changes in the starch, such as gelatinization and retrogradation [163,164,165,166]. In terms of the characterization of cassava starch, the hydroxyl group protons appear in the ^1^H NMR spectrum around 4–6 ppm, and are easily distinguishable (Figure 7A); however, the protons on the carbohydrate ring appear as two overlapping signals in the range of 4–3 ppm [167]. In the ^13^C NMR spectrum, the representative peaks are attributed to the carbons: C1 (90–105 ppm), C2, C3, C5 (65–78 ppm), C4 (77–88 ppm), and C6 (56–64 ppm) (Figure 7B) [168,169]. Furthermore, NMR is an important tool for determining structural characteristics, conformational changes, and chemical modifications in cassava starch. For example, ^1^H NMR can be used to determine the branching ratios of cassava starch (α-1,4)/(α-1,6) and to quantify the amylose/amylopectin (AM/AP) content. This is possible by integrating the (α-1,4) C1 proton and (α-1,6) C1 proton signals in the NMR spectra carried out at 90 °C [170]; this methodology has also been used in other research [171].

Proton NMR has been reported as a tool to elucidate the chemical structure of cassava starch and to reveal chemical modifications. Cassava starch and cassava starch chemically modified by grafting with 2-ethylhexyl acrylate (EHA) were analyzed by ^1^H NMR in the liquid state, using deuterated dimethyl sulfoxide (d6-DMSO) for both starch and starch-g-PEHA at 100 °C [136]. The chemical modification increased the hydrophobicity of the cassava starch. Additionally, there are reports using d6-DMSO at a lower temperature (50 °C) [172]. Moreover, ^1^H NMR has been used to determine chemical modifications, such as hydroxypropylation, by employing the α-amylase (α-AM) digestion of cassava starch to increase the concentration of the analyte, and subsequently to analyze the resulting dextrins [173]. Other chemical modifications of cassava starch characterized by ^1^H NMR and/or ^13^C NMR include acid hydrolysis [168], esterification with maleic anhydride [172], and reactions with diethyl maleate, dipropyl maleate, and dibutyl maleate [64], as well as modification with acrylic acid monomer [174], poly (ethylene glycol) 4000 and propylene oxide [175], formation of carboxymethylated cassava starch (CMS) [176], methylation with dimethyl carbonate [177], succinylation [178], octenylsuccinylation of oxidized cassava starch [179], and complexation of cassava starch with ferulic acid [180].

For chemical modifications of cassava starch, ^1^H NMR and/or ^13^C NMR are important techniques to determine the degree of chemical substitution, as demonstrated by Cuenca et al., who determined the degree of acetylation by integrating the ^13^C NMR signals in the spectrum [171]. Furthermore, these techniques allow for the determination of chemical and molecular interactions between cassava starch and other components, such as the complexation of α-amylase-hydrolyzed cassava starch with zinc [181]. In addition, ^13^C CP/MAS solid-state NMR enables the identification of the C1 resonance of glucose units, which contains information regarding the crystalline nature of starch and allows for monitoring physical transitions [182,183]. The gelatinization process has been studied by observing the disruption of the molecular order within starch granules during the process, where the residual molecular structure of the starch was analyzed by ^13^C CP/MAS NMR. The results indicated that both molecular and crystalline orders are disrupted during gelatinization. These findings were compared with the residual gelatinization enthalpy determined by DSC, revealing that the enthalpy change observed during gelatinization mainly reflects the loss of double-helical organization in starch [184]. Data from ^13^C CP/MAS NMR can also be correlated with FT-Raman spectroscopy and XRD to analyze cassava starch samples with varying levels of crystallinity after different hydrothermal treatments, where the crystalline-phase contents determined by NMR were found to be close to those determined by XRD [185]. A similar correlation strategy was employed to analyze the crystallinity of cassava starch hydrolyzed with 6% (*w*/*v*) HCl at 25 °C over different reaction times (0, 12, 24, 48, 96, 192, 384, and 768 h). Carbon-13 CP/MAS NMR revealed that acid hydrolysis increases the double helix content and decreases the amorphous region of cassava starch [182].

NMR has also been applied to the identification of non-digestible polysaccharides in starch, such as cellulosic glucans. In these analyses, samples are often treated with NaOH, which acts as a chaotropic agent, enabling non-specific enzymes to better access resistant starch and promote the breakdown of glycosidic bonds [185]. Sluiter et al. (2021) used ^13^C cross-polarization magic-angle spinning (CPMAS) solid-state NMR to characterize residual cellulose after alkaline treatment [186]. The characteristic signals observed at 89 ppm and 105 ppm indicated that the molecular structure of the cellulose remained intact following treatment [186]. NMR can also be coupled with mass spectrometry to obtain detailed structural information about modified starches and cellulose, including the degree and position of molar substitution on the polymer backbone [187]. However, NMR is more commonly used to gather qualitative rather than quantitative data for these biopolymers [188,189].

### 4.3. Raman Spectroscopy

Raman spectroscopy is a powerful technique used to analyze and evaluate the chemical environment surrounding individual atoms, with wide applications in the study of synthetic polymers [190], proteins [191], polysaccharides [192], and lipids [193], among others. The Raman spectra provide information about the vibrational motions of molecules, where the vibrational frequencies depend on the molecular geometry and chemical composition [194]. For cassava starch, Raman spectroscopy has been used to characterize both the native starch and its chemical modifications. Three main spectral regions can be explored: (i) Region I: 3600–3000 cm^−1^, corresponding to O–H stretching vibrations; (ii) Region II: 3000–2800 cm^−1^, related to C–H stretching vibrations; (iii) Region III: 1600–200 cm^−1^, known as the fingerprint region [195]. A typical Raman spectrum of cassava starch was reported by Almeida et al. (2010) [196]. The C–H and O–H stretching vibrations associated with the AP and AM structures are clearly identifiable in Regions I and II. By contrast, in Region III, the fingerprint region exhibits significant signal overlap, which complicates the assignment of individual bands. Nevertheless, in the spectra reported by Almeida et al. (2010) [196], it is possible to observe distinct vibrational modes of the glucose pyranose ring at 440, 478, 576, 673, and 710 cm^−1^. The α-1,4-glycosidic bonds are identified by a band near 940 cm^−1^, corresponding to the C–O–C stretching vibration [197]. A band around 1262 cm^−1^ is associated with the CH_2_OH group vibration of AM. Bending vibrations of the C–O–H groups are observed near 1127 and 1339 cm^−1^. Additionally, CH_2_ twisting, scissoring, and bending vibrations appear at approximately 1339, 1380, and 1461 cm^−1^ [195,198]. An important feature of both the FTIR and the Raman spectra is the presence of water absorbed in the amorphous regions of cassava starch, which typically appears in the range of 1500–1750 cm^−1^ [195,198].

Raman spectroscopy allows for the identification of chemical modifications in the structure of cassava starch. The implementation of a chemometric analysis to process the Raman data has proven useful for identifying chemical modifications or treatments applied to cassava starch. Dupuy and Laureyns used Raman spectroscopy to identify the type of chemical modification in cassava starch (hydroxypropyl and acetyl substitution) by applying principal component analysis (PCA) and partial least squares (PLS), finding this to be an effective tool for classifying and identifying chemical modifications in cassava starch [199]. PCA and PLS analyses have also been used to identify cassava starches irradiated with gamma rays—a process employed to extend shelf life or improve food safety by reducing microorganisms. This research found that the water signal provided better classification of the irradiated starches [198]. Furthermore, PCA has been applied to determine the AM content and to detect adulteration in cassava starch using Raman spectroscopy, owing to changes in band intensity around 470 cm^−1^ that indicate variations in the AM content [196].

Using the spectral bands, it is also possible to observe conformational changes in cassava starch. According to Mutungi et al. (2012) [185], there is a strong linear correlation between crystallinity and Raman signals, especially for the mode around 480 cm^−1^, where the intensity depends on the polymer chain length [18,185]. Moreover, Raman spectroscopy combined with chemometric analysis has been used to detect cassava starch adulterated with wheat flour. The chemometric models demonstrated high sensitivity (87.1%), specificity (86.8%), and accuracy (86.9%) in predicting adulterated samples [200]. However, to our knowledge, although researchers have used Raman spectroscopy to analyze the gelatinization and retrogradation processes in starches from various sources [201,202,203], there are still few studies focused specifically on cassava starch.

## 5. Conclusions

Cassava root is an important food source of carbohydrates in tropical regions with a production of ~270 million tons around the world, and provides ~73.7% to 84.9% (dry weight) of starch, which is a co-polymer chain of AM (13–29.5%) and AP (70–87%). In addition, this biopolymer is biodegradable and cheap, making attractive raw material for manufacturing with and without physical and chemical modifications. Thus, it is necessary for the physicochemical characterization of cassava starch to determine the factor that affects its physical and chemical properties, allowing for an understanding of its behavior and industrial application. The cassava starch granules have different shapes including truncated, oval, round, spherical, irregular, and polygonal shapes, which present difficulties to disperse aqueous solution without affecting the AM and AP structure. Several analytical methods have been used to determine the M_w_, R_g_, and R_H_ parameters of the cassava starch; however, there are wide differences between the values reported in the literature, demonstrating that it is a complex system and depends on innumerable factors, including the growth parameters of the crop, environmental condition, and genetic factors. Also, these parameters affect the behavior of the cassava starch granule and protect the polymer from physical changes, i.e., gelatinization, pasting, and retrogradation. The spectroscopy chemical characterization of cassava starch has been well established by NRM, FTIR, and Raman spectroscopy, even allowing for the development of analytical methods to quantify this carbohydrate, and to make evident the adulterations of its flours.

## Figures and Tables

**Table 1:** Chemical Composition of Cassava Starch.

Crop	Amylose (%)	Protein (%)	Lipid (%)	^a^ Fiber (%)	Ref.
Rayong 9	-	4.85 ± 0.09	0.08 ± 0.01	3.44 ± 0.17	[10]
Rayong 11	-	4.55 ± 0.06	0.16 ± 0.01	3.14 ± 0.26	[10]
Cultivar TMS 30470	29.5 ± 0.67	0.32 ± 0.01	0.17 ± 0.00	1.2 ± 0.00	[23]
Rayong 1	24.1	0.17 ± 0.04	-	-	[24]
KU 50	21.4	0.30 ± 0.04	-	-	[24]
N.R	23.0	0.1	1.2	-	[25]
N.R	23.0	0.1	-	-	[25]
N.R	16.11 ± 1.0	0.31 ± 0.01	0.20 ± 0.03	0.23 ± 0.06	[26]
HMC-1	-	0.26	0.31	-	[27]
MH97/2962	-	0.47	0.55	-	[28]
TMS 326	-	1.26 ± 0.06	1.59 ± 0.13	1.95 ± 0.08	[29]
TME 419	-	0.51 ± 0.08	0.94 ± 0.16	2.15 ± 0.14	[29]
M82/00032	24.0 ± 0.00	1.75 ± 0.00	-	1.54 ± 0.21	[30]
I93/0560	13.2 ± 0.00	1.74 ± 0.00	-	2.04 ± 0.12	[30]
N.R	23.65 ± 0.15	0.14 ± 0.22	0.11 ± 0.01	-	[31]
Adira-4	26.3	-	0.013	-	[32]
TMS 98/0581	26.41 ± 0.13	0.40 ± 0.07	0.13 ± 0.001	-	[33]
ICA-C523-7	14.67 ± 0.25	0.60 ± 0.02	0.31 ± 0.00	0.04 ± 0.01	[34]
MBra 383	14.43 ± 0.51	0.60 ± 0.03	0.32 ± 0.01	0.05 ± 0.01	[34]
N.R	17.2 ± 0.4	1.9 ± 0.2	-	2.8 ± 0.3	[35]

(-) = Not reported. ^a^ Fiber corresponds to crude fiber.

**Table 2:** Cassava starch content (% of dry weight) and granule morphology from different regions around the world.

Crop	Age (Months)	Region	Starch Content (% Total Cassava)	Size (Average, µm)	Shape	Ref.
Rayong 1 (1957)	16	Thailand	-	8–22	oval, round, and truncated	[24]
TMS 98/0581	10	Nigeria	87.44–89.64	6–22	irregular, truncated, and oval-shaped granules	[33]
Rayong 90	12	Thailand	-	5–25	Not described	[38]
Rayong 9	12	Thailand	81.3	7–30	Not described	[39]
Crops-2017	11	Brazil	86.9	16–20	circular with some truncated	[40]
-		Thailand	95	10–40	truncated	[41]
Kasetsart 50	12	Thailand	-	9–22	spherical and some were truncated	[42]
-	-	-	-	11.9	rounded or oval shape	[43]
CMR 38-125-77	9	Thailand	82.16	7–29	Not described	[39]

**Table 4:** DSC parameters of the gelatinization process of native cassava starch from various studies reported in the literature.

T–Initial (°C)	Tp–Peak (°C)	T–Final (°C)	ΔH (J g^−1^)	Ref.
62.4	69.3	84.1	4.8	[95]
66.9 ± 0.2	70.1 ± 0.1	85.1 ± 0.7	15.6 ± 0.5	[96]
58.6 ± 0.2	63.3 ± 0.4	69.2 ± 0.5	13.8 ± 0.3	[97]
63.9 ± 0.2	70.5 ± 0.1	82.7 ± 1.0	8.5 ± 0.1	[98]
64.3 ± 0.0	71.7 ± 0.0	81.0 ± 0.0	17.4 ± 0.1	[99]
55.1 ± 1.9	70.2 ± 0.3	80.1 ± 0.9	16.3 ± 0.7	[100]
64.0 ± 0.0	70.2 ± 0.1	78.3 ± 0.1	12.0 ± 0.1	[41]
60.2 ± 0.1	67.5 ± 0.0	75.83± 1.0	13.5 ± 0.0	[101]

**Table 5:** FTIR spectroscopy data of starch from different sources (cassava, corn, and potato).

Functional Groups	Vibration	Wave Number (cm^−1^)		
		Cassava	Corn	Potato
O–H	Stretching	3448	3448	3523
C–H	Stretching	2930	2929	2927
CH_2_	Symmetric deformation	1437	1437	1437
CH_2_	Symmetric scissoring	1417	1415	1419
C–H	Symmetric bending	1381	1381	1381
C–O–C	Asymmetric stretching	1157	1157	1157
C–O	Stretching	1082, 1016	1082, 1018	1082, 993
C–O–C	Ring vibration of carbohydrate	929, 860, 763	929, 860, 763	929, 860, 763

Data reported by [138,139,143].

## Data Availability

The original contributions presented in this study are included in the article. Further inquiries can be directed to the corresponding authors.

## Abbreviations

The following abbreviations are used in this manuscript:

AM	amylose
AP	amylopectin
SEM	scanning electron microscopy
AFM	atomic force microscopy
M_w_	molecular weight
R_g_	apparent radius of gyration
R_H_	apparent hydrodynamic radius
SEC	size-exclusion chromatography
GPC	gel permeation chromatography
HPSEC	high-performance size exclusion chromatography
MALLS	multi-angle laser light scattering
DRI	differential refractive index
A4F	asymmetrical flow field-flow fractionation
HPLC	high-performance liquid chromatography
DMSO	dimethyl sulfoxide
PDI	polydispersity index
D_t_	translational diffusion coefficients
XRD	X-ray diffraction
DSC	differential scanning calorimetry
T_g_	glass transition temperature
T_m_	melting endotherm
ΔH	enthalpy change
RVA	Rapid Visco Analyzer
NaCl	sodium chloride
FTIR	Fourier-transform infrared spectroscopy
NMR	nuclear magnetic resonance spectroscopy
PCA	principal component analysis
PLS	partial least squares
